# In Vivo MRI Measurement of Spinal Cord Displacement in the Thoracolumbar Region of Asymptomatic Subjects with Unilateral and Sham Straight Leg Raise Tests

**DOI:** 10.1371/journal.pone.0155927

**Published:** 2016-06-02

**Authors:** M. Rade, M. Könönen, J. Marttila, M. Shacklock, R. Vanninen, M. Kankaanpää, O. Airaksinen

**Affiliations:** 1 Kuopio University Hospital, Department of Physical and Rehabilitation Medicine, Kuopio, Finland; 2 Josip Juraj Strossmayer University of Osijek, Faculty of Medicine, Orthopaedic and Rehabilitation Hospital “Prim. dr.Martin Horvat”, Rovinj, Croatia; 3 Kuopio University Hospital, Department of Radiology, Kuopio, Finland; 4 Neurodynamic Solutions, Adelaide, Australia; 5 Tampere University Hospital, Department of Physical and Rehabilitation Medicine, Tampere, Finland; University of Szeged, HUNGARY

## Abstract

**Background:**

Normal displacement of the conus medullaris with unilateral and bilateral SLR has been quantified and the "principle of linear dependence" has been described.

**Purpose:**

Explore whether previously recorded movements of conus medullaris with SLRs are i) primarily due to transmission of tensile forces transmitted through the neural tissues during SLR or ii) the result of reciprocal movements between vertebrae and nerves.

**Study design:**

Controlled radiologic study.

**Methods:**

Ten asymptomatic volunteers were scanned with a 1.5T magnetic resonance (MR) scanner using T2 weighted spc 3D scanning sequences and a device that permits greater ranges of SLR. Displacement of the conus medullaris during the unilateral and sham SLR was quantified reliably with a randomized procedure. Conus displacement in response to unilateral and sham SLRs was quantified and the results compared.

**Results:**

The conus displaced caudally in the spinal canal by 3.54±0.87 mm (mean±SD) with unilateral (*p*≤.001) and proximally by 0.32±1.6 mm with sham SLR (*p*≤.542). Pearson correlations were higher than 0.99 for both intra- and inter-observer reliability and the observed power was 1 for unilateral SLRs and 0.054 and 0.149 for left and right sham SLR respectively.

**Conclusions:**

Four relevant points emerge from the presented data: i) reciprocal movements between the spinal cord and the surrounding vertebrae are likely to occur during SLR in asymptomatic subjects, ii) conus medullaris displacement in the vertebral canal with SLR is primarily due to transmission of tensile forces through the neural tissues, iii) when tensile forces are transmitted through the neural system as in the clinical SLR, the magnitude of conus medullaris displacement prevails over the amount of bone adjustment.

## Introduction

As previously suggested [1], physical tests for nerve root tension signs have been designed to aid the diagnosis of intervertebral disc (IVD) herniation causing lumbar radiculopathy. Of those, the straight leg raise (SLR) test is the most widely used, reliable and valid in detecting intervertebral disc herniations verified at surgery [2–5].

It is generally accepted that the SLR tests the mechanosensitivity of the L5 and S1 nerve roots by means of lifting the patient’s lower limb with an extended knee which applies tension to the related neural structures. However, the precise mechanical effects on the nerve roots in response to this test are still a matter of debate between researchers.

It is known that, even though in varying amounts, the lumbosacral nerve roots move distally (between a minimum of 0.5 mm and a maximum of 10 mm) depending on the study and methods, which include cadavers and patients at surgery [4–11]. It appears that the neuromechanical effects are due to distal application of tension through the hip, knee and ankle producing elongation of the nerve bed in the lower limb [12]. Also, contributing to this elongation, knee extension produces distal movement of the sciatic nerve in the thigh [13].

Although the SLR is popular and with it there exist small variations in the amount of movement of the nerve roots between studies, there is consensus in the direction of movement which is caudal [4–8,11,14–17].

Even though the magnitude of nerve root excursion with the SLR is not entirely consistent between studies, it has become important for measurements of spinal cord excursion to be made. This is because, now that normal baseline measurements have been made [1,18], we are in a position to measure any deviations from normal in symptomatic populations, hence potentially contributing to radiological diagnostic efficacy.

Rade and colleagues demonstrated conus medullaris caudal displacement of 2.33± 1.2 mm with the unilateral SLR and 4.58±1.48 mm with the bilateral SLR, both tests to 50° hip flexion [1,18]. Moreover, at 60° SLR the conus caudal displacement was shown to increase, with 3.54 ± 0.87 mm of caudal displacement with unilateral SLR and 7.42 ± 2.09 mm with bilateral SLR [19]. In both studies it emerged that the magnitude of conus medullaris displacement with unilateral SLR was doubled with the bilateral SLR. This phenomenon was explained by the “principle of linear dependence” in which, due to the structural continuum of neural tissues in which the lumbosacral neural roots are directly connected via the cauda equina to the spinal cord, and in which the tensile forces can be transmitted throughout these neural structures and the dura, the magnitude of conus medullaris displacement is proportional to the displacement of L5 and S1 nerve roots and dependent on the number of nerve roots involved into the movement (i.e. unilateral and bilateral SLRs).

However, in their original investigation, Rade and colleagues did not provide a conclusive answer to the question of whether it is the conus medullaris that displaces caudally within the spinal canal in response to a SLR, or it is actually the spinal canal that adjusts around the spinal cord in response to the pelvis motion during SLR.

**Fig 1:**
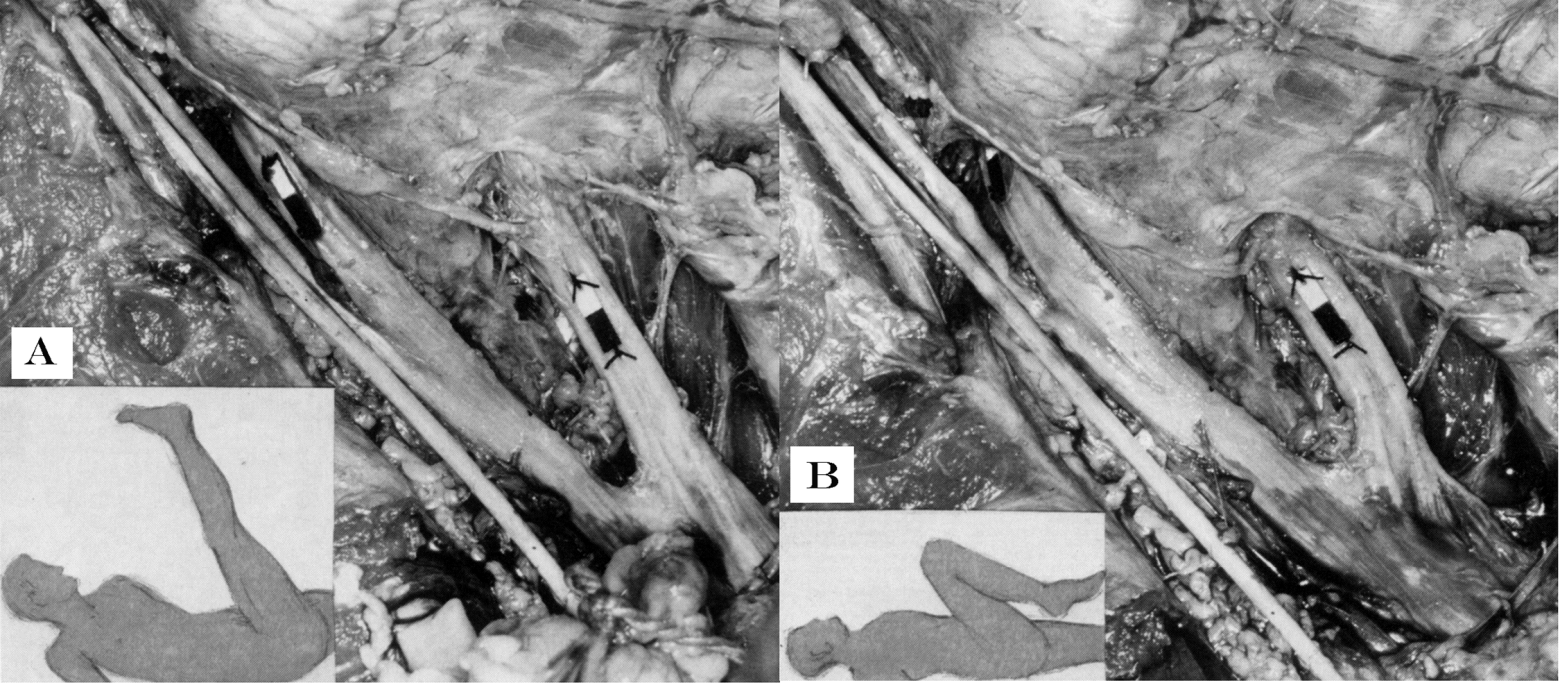
Breig’s cadaver investigations showing the effects on L5 and S1 nerve roots within and outside the foramina with A) SLR and B) Sham SLR with hip and knee flexion. Paper marks are sutured to the epineurium of the spinal nerves. It is easily noticeable that the paper marks are drawn further from the foramina as a result of transmission of tensile forces distally along the sciatic nerve (A). This does not occur in B with the Sham SLR when the paper marker of L5 nerve root instead rests inside the L4-5 foramen. From Shacklock M: Biomechanics of the Nervous System: Breig revisited. Adelaide: Neurodynamic Solutions NDS; 2007, with permission.

In 1978 Breig documented significant differences in mechanical responses of L5 and S1 nerve roots when the SLR was performed with the knee flexed [20,21]. Nearly no displacement was found with a Sham SLR, with the accepted explanation being that, as the knee flexion movement did not allow the sciatic nerve to be tensioned and pulled distally by the maneuver, tensile forces were not transmitted along the nerves [20,21] (Fig 1).

Following this, we decided to replicate and build upon Breig’s experiments using magnetic resonance imaging to confirm the results of the Breig studies and understand whether the behavior of conus medullaris in response to a Sham SLR in in-vivo and structurally intact asymptomatic human subjects would be similar to the one described in the lumbar nerve roots by Breig in cadavers. Using the same methods as in our previous study [11] of in vivo measurement of spinal cord displacement with the unilateral straight leg raise (SLR) in asymptomatic subjects, we further measured cord displacement during the passive sham SLR, comparing these data with those of the unilateral genuine SLR [11].

The analysis of such responses was to provide an answer to the question of whether previously recorded movements of conus medullaris with the unilateral and bilateral SLR [1,18,19] are i) primarily due to transmission of tensile forces transmitted through the neural tissues during SLR or ii) the result of reciprocal movements between vertebrae and nerves.

## Materials and Methods

### Subjects

Following the results of a pilot study considering the dimensions of the scanner (Siemens Magnetom Aera 1.5T, Erlangen, Germany), it was calculated that subjects taller than 183cm were needed to allow performance of the SLR in the scanner, permitting at least 60° of unrestricted hip flexion [19].

As before [19], a total of 11 volunteers were recruited and screened for eligibility, one of whom met the exclusion criteria (Table 1) and was thus excluded from the study. Ten asymptomatic volunteers ranged from 20 to 32 years (mean age 25.1±3.9 years), height 186.7± 2.9 cm, BMI 24.22±3.92, were included in the study.

**Table 1:** Exclusion and inclusion criteria.

**Exclusion criteria**
• Subjects currently experiencing painful symptoms in the tested area
• Incomplete and/or painful knee extension
• Incomplete and/or painful hip range of motion
• History of known neurological disorders of the tested extremity
• History of diagnosed lumbar intervertebral disc herniation
• History of previous abdominal or lumbar surgeries
• Other joint involvement, like arthritis or already recognized metabolic bone disease
• Subjects with any known arthrogenic, muscular or neurogenic dysfunctions in the lumbar spine area which, on provocative physical testing, gave positive signs and/or pain into the lower limb
• Presence of pacemakers and ferromagnetic implants
**Inclusion criteria:**
• Subjects assessed to be asymptomatic
• Subjects’ consent to participation by signing the consent form
• No present exclusion criteria at the time of testing
**Summary of exclusion criteria:** *All the volunteers were screened to be asymptomatic and to have a pain-free and complete range of bilateral movement in the hip*, *knee and ankle joints and did not match the exclusion criteria*.

In accordance to earlier studies [1,18,19], asymptomatic volunteers were chosen in order to make use of the normal situation providing normative measurements and avoid potentially confounding variables such as local impairments or neural dysfunctions that may occur in a symptomatic population.

**Fig 2:**
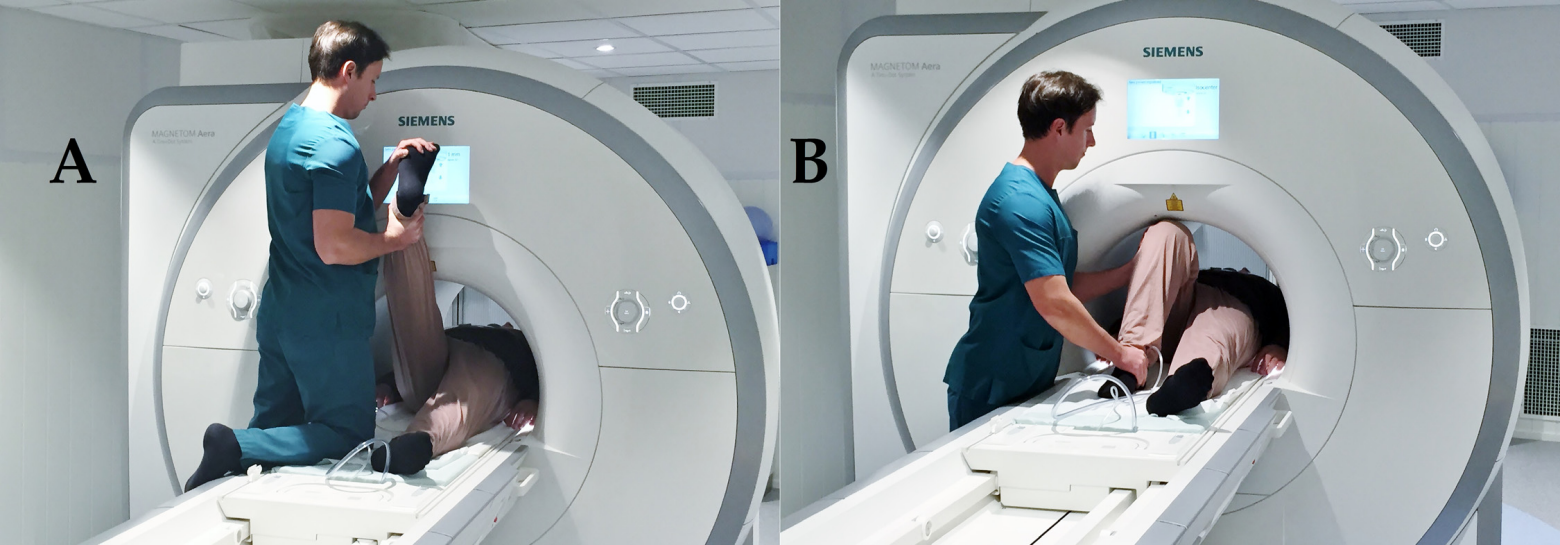
Passive right SLR (A) and right Sham SLR (B) with subject lying supine in the magnetic resonance scanner.

All aspects of work that involved human patients was conducted with the ethical approval of the Research Ethical Committee of Kuopio University Hospital, approval number 79/2012. All tested subjects signed an informed consent form and the study was performed in accordance with the Declaration of Helsinki. The individual in this manuscript, Fig 2, has given written informed consent (as outlined in PLOS consent form) to publish their picture.

### Devices

As in previous experiments [11], subjects lay in supine in a 1.5T magnetic resonance (MR) scanner (Siemens Magnetom Aera, Erlangen, Germany). The imaging area was centered approximately 3cm proximally from the xiphoid process of the sternum and the coronal images centered at the lower part of the imaging area to T12-L2 anatomic region. The volunteers were scanned using a 32-channel spine matrix coil.

Different scanning sequences for planning and for measurement were used.

1. Planning: T2 weighted turbo spin echo sequence (TR 3530ms, TE 96ms, 17 slices, slice thickness 3mm, FOV 300mm, in plane resolution 0.8x0.8mm, flip angle 150 degrees). Sagittal slices were aligned with the spinal cord to allow better identification of the conus medullaris.
2. Measurement: T2 weighted spc 3D-sequence (TR 1800ms, TE 128ms, slice thickness 1mm, sagittal scan, FOV 300mm, phase encoding direction proximal to caudal, in plane resolution 0.6x0.6mm, flip angle 160 degrees).

Coronal, axial and sagittal slices (slice thickness 1mm, approximately 70 slices in each plane) were reconstructed from the native T2 weighted spc 3D-sequence sagittal scans using the MPR program available in Sectra PACS workstation (Sectra Workstation IDS7, version 15.1.8.5–2013 –Sectra AB, Sweden).

### Subject positioning and test movements

It was explored whether any difference in conus medullaris displacement would occur between the sham and genuine SLR. The Sham SLR was performed with the flexed knee as in Breig’s cadaver experiments [20,21], so as to take the tension off the nerve roots while allowing pelvic motion (posterior rotation) and natural concurrent adjustment of the spinal canal as during the genuine, standard, SLR (reduction of the lordosis).

The volunteers were scanned in the following positions in a random order:

- Neutral: Subject lying supine, aligned symmetrically in the anatomic position, lower limbs extended and relaxed.
- Right SLR: passive SLR to maximum hip flexion allowed by the MR scanner (60°—hip flexion was taken to the point of contact with the gantry of the machine), holding the lower limb still, with the knee extended and the ankle in a plantargrade position (0° of dorsiflexion), (Fig 2).
- Left SLR: passive SLR as with the right side.
- Sham right: SLR to the same degree of hip flexion as during the right SLR. However, as in Breig [20,21] this time the subjects’ knee was flexed (Fig 2).
- Sham left: passive SLR with flexed knee as with the Sham test on the right side.

Due to the MR device architecture with a tube diameter of 70cm a mean value of 58.1±4.4° (Mean±SD) of hip flexion could be achieved.

As before [1], hip flexion was measured with an oil-filled precision goniometer placed on the anterior surface of the distal third of the tibia. This method has been shown to have good intra-observer reliability with the SLR [22] and was considered safe to be operated in the MR scanning room, security zone IV.

Each movement was performed twice for evaluation of reliability. Three practitioners performed the maneuvers in a random sequence in order to avoid possible series effects.

Cervical flexion in the subjects was always avoided so as not to influence spinal cord position or movement.

### Conus medullaris displacement measurement

**Fig 3:**
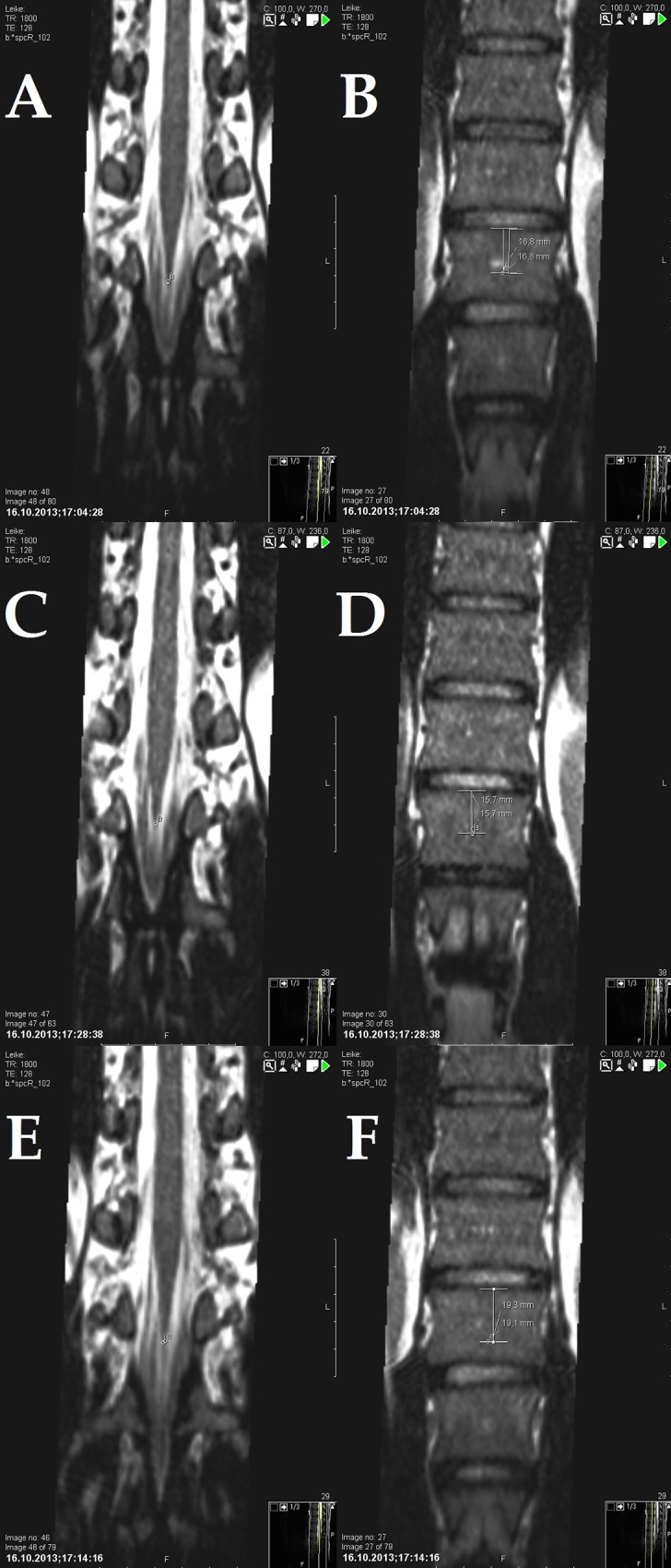
Magnetic resonance scans. Coronal slices of the thoracolumbar region of a sample subject during A. reference scan, C. Sham SLR, E. Unilateral SLR are presented. The apex of the conus medullaris is marked. The vertical distances from the upper intervertebral surface of the adjacent vertebral body are marked and presented in B, D, and F. Different measurements from both observers are presented. Note the conus medullaris displacement being proximal with Sham SLR (C, D).

The displacement of the conus medullaris relative to the upper intervertebral surface of the adjacent vertebra during the unilateral passive right, left SLR and unilateral right, left Sham SLR was quantified and compared with the position of the conus in the neutral (anatomic) position (Fig 3 ABCDEF).

Consistently with earlier investigations [19], measurements were taken twice by the main author, with two months between each measurement, and once by co-author (JM) in order to allow for evaluation of intra- and inter-observer reliability.

The two observers independently assessed the conus displacement by first identifying the tip of the conus. The tip was initially identified on the coronal slices and its position concurrently verified on the axial and sagittal slices using the crosshair and localizer tools available in Sectra PACS workstation. Particular care was taken to identify the origin of filum terminale so as to confirm the location of the tip of the conus.

The mark on the tip of the conus was then precisely projected at the center of the adjacent vertebral body by using the crosshair and localizer tools available in Sectra PACS program (Sectra Workstation IDS7, version 15.1.8.5–2013 –Sectra AB, Sweden). As in Rade et al. [1,18,19] the distance between the mark on the vertebral body and the anatomical reference point represented by the upper intervertebral surface was measured on the coronal slices. The measurements were made using Sectra PACS program (Sectra Workstation IDS7, version 15.1.8.5–2013 –Sectra AB, Sweden).

All the presented metric values were truncated to the next lowest decimal integer (3.55 = 3.5) to provide more conservative and reliable data.

### Statistical methods

As in previous investigations [1,18,19], the purpose of the data analysis was to detect any statistically significant differences in medullar cone position between the reference and test positions for the right SLR, left SLR, and right and left Sham SLRs.

A two-tailed hypothesis that the conus would displace in response to SLRs versus no change was tested.

As in previous investigations [1,18,19], Pearson correlation between the displacements found in two scans of the same maneuvers performed on each subject was calculated as well as for inter- and intra-observer reliability.

Having found strong correlation between the measures from different scans of the same maneuvers performed on each subject, as well as high correlations between different measurements performed by different observers on those scans, it was decided to average all the available measurements when presenting the mean values and their standard deviations, in order to present the results as accurately and conservatively as possible.

Student´s t-test was used to test the significance of medullar displacement during SLR maneuvers in relation to the position found in the reference scans. The Alpha level was set at *p* < .05.

Owing to the relatively small data sample (N = 20) it was postulated that the data distribution is leptokurtic, thus 95% Confidence Intervals (95% CI) were calculated using t distribution.

The Observed Power was calculated on the data using t distribution, while the minimum number of subjects needed to extract statistically significant results was calculated from the collected data. Statistical analysis was performed using R Program (R Foundation for Statistical Computing, Vienna, Austria), Version 2.15.2 (2012).

## Results

The number of subjects required to produce statistically significant results (*p* < .05) was 2621 for right Sham SLR and 78 for left Sham SLR, and three for both right and left unilateral SLR.

When compared to the position in the neutral (anatomic) position, the conus medullaris displaced caudally in the spinal canal by 3.57 ± 1.14 mm (Mean±SD) (95% CI -4.27, -2.87) with the left SLR (*p*≤.001) and 3.52 ± 0.77 mm (95% CI -4.17, -2.87) with the right SLR (*p*≤.001).

**Fig 4:**
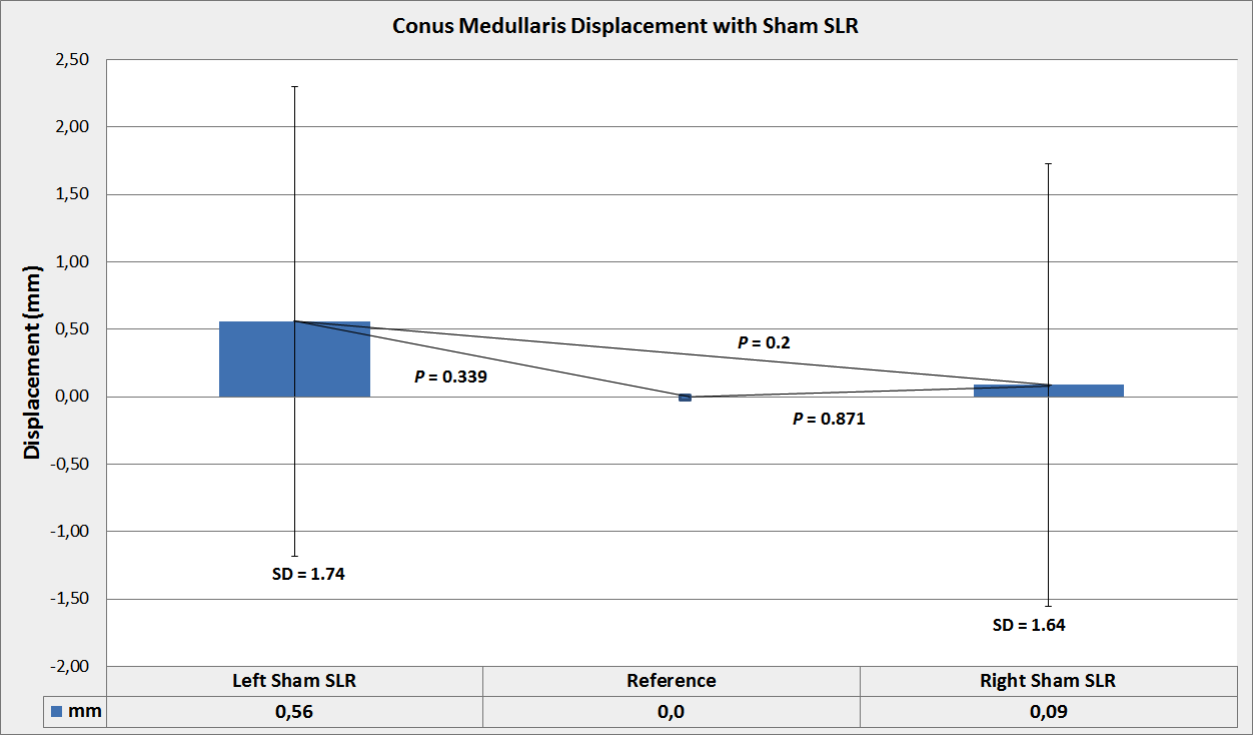
Conus medullaris displacement with unilateral Sham SLR test. The mean value and standard deviations of measurements are presented. Statistical significance was not achieved for any of these comparisons made; reference versus right Sham SLR, reference versus left Sham SLR, right Sham SLR versus left Sham SLR. Values are expressed as positive to indicate proximal displacement of the conus medullaris relative to the upper intervertebral surface of the adjacent vertebral body.

On the other hand, conus medullaris displacement in the thoracolumbar canal with the both Sham SLRs was proximal, and this by 0.09± 1.64 mm (95% CI -0.80, 0.97) with the right Sham SLR (*p* = .871) and 0.56± 1.74 mm (95% CI -0.35, 1.47) with left Sham SLR (*p* = .339) (Fig 4).

**Fig 5:**
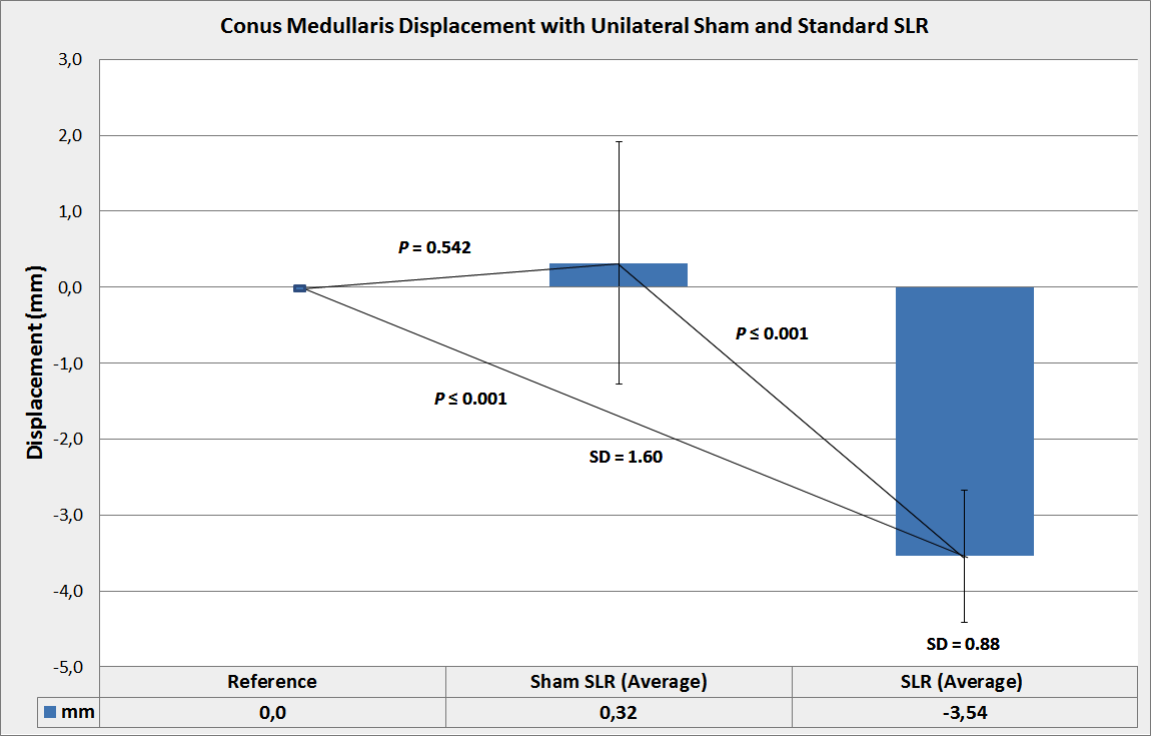
Conus medullaris displacement with unilateral Sham SLR and standard SLR. Mean value and standard deviations of measurements are presented. Values are expressed as positive or negative to indicate respectively proximal or caudal direction of conus medullaris displacement. Even if the average value indicates mean conus displacement in response to a Sham SLR as proximal, the conus displaced in both proximal and distal direction. This showed to be consistent in terms of direction and magnitude within subjects, but not between subjects.

Comparison between right and left Sham SLR did not show statistically significant difference (*p* = .2) (Fig 4). Strong statistical significance was achieved for comparisons between unilateral left SLR and left Sham SLR (*p*≤.001) and unilateral right SLR and right Sham SLR (*p*≤.001) (Fig 5).

The Pearson correlations, as well as observed ranges and results for the observed power are presented in Tables 2 and 3.

**Table 2:** Reliability values for medullar cone displacement measurements with unilateral SLR with extended and flexed knee. Pearson’s correlations are presented.

	RESULTS RELIABILITY	INTRA OBSERVER	INTER OBSERVER
**RIGHT SLR**	0.990	0.999	0.999
**LEFT SLR**	0.996	0.999	0.999
**RIGHT SHAM SLR**	0.991	0.999	0.999
**LEFT SHAM SLR**	0.989	0.999	0.996
**REFERENCE SCAN**	0.995	0.999	0.998

**Table 3:** Ranges of medullar cone displacement with unilateral SLR with extended and flexed knee along with observed power.

	MIN (mm)	MAX (mm)	*p* VALUES	NUMBER OF SUBJECTS TESTED	NUMBER OF SUBJECTS NEEDED FOR SIGNIFICANT RESULTS	OBSERVED POWER
**RIGHT SLR**	-2.3	-4.9	≤.001	10	3	1
**LEFT SLR**	-2.1	-5.4	≤.001	10	3	1
**RIGHT SHAM SLR**	-1.7	+3.8	.871 *	10	2621	0.054
**LEFT SHAM SLR**	-1.7	+3.1	.339 *	10	78	0.149

Note: (+) and (–) signs are used to mark proximal and caudal displacement of conus medullaris relative to the upper intervertebral surface of the adjacent vertebra in response to SLRs.

* indicates a not significant difference.

## Discussion

Generally—the key objective of this study was to, using a comparison between genuine and sham SLR, ascertain if there is evidence expressed in the movement of the spinal cord to show that the emphasis or dominance of the mechanism of the genuine SLR is through the neural tissues, in comparison to bone movement.

More specifically, in the present study, the authors sought to confirm the results of the Breig studies [20,21] using MRI and so investigate in vivo conus medullaris displacement in the thoracolumbar vertebral canal during both the passive unilateral SLR and passive sham SLR in asymptomatic subjects in order to answer whether previously recorded movements of conus medullaris in response to unilateral and bilateral SLRs [1,18,19] are i) primarily due to transmission of tensile forces transmitted through the neural tissues during SLR ii) or a result of reciprocal movements between vertebras and nerves, or iii) a combination of both.

Consistent with previous work [19], the spinal cord displaced caudally by an average of 3.54 ± 0.87 mm in response to the genuine SLR test with 60° of hip flexion, and by an average of 0.32±1.6 mm with the Sham SLR to the same range of hip flexion as with the genuine unilateral SLR, indicating that the conus displaces significantly less in absence of tensile forces transmitted through the lumbosacral nerve roots and dura, as with the Sham SLR.

Interestingly, contrary to what was measured with unilateral SLRs, the mean values collected during Sham SLR showed minimal conus medullaris displacement which was in the proximal direction instead of distal. It is of interest to highlight that, in this study, conus medullaris displacement in response to Sham SLR occurred indiscriminately in both caudal and proximal direction relative to the adjacent vertebral body, and showed to be very consistent in terms of direction and magnitude within subjects, but not between subjects. This means that in some subjects the conus displaced always proximally and in some always distally by the same magnitude every time the maneuver was performed.

**Fig 6:**
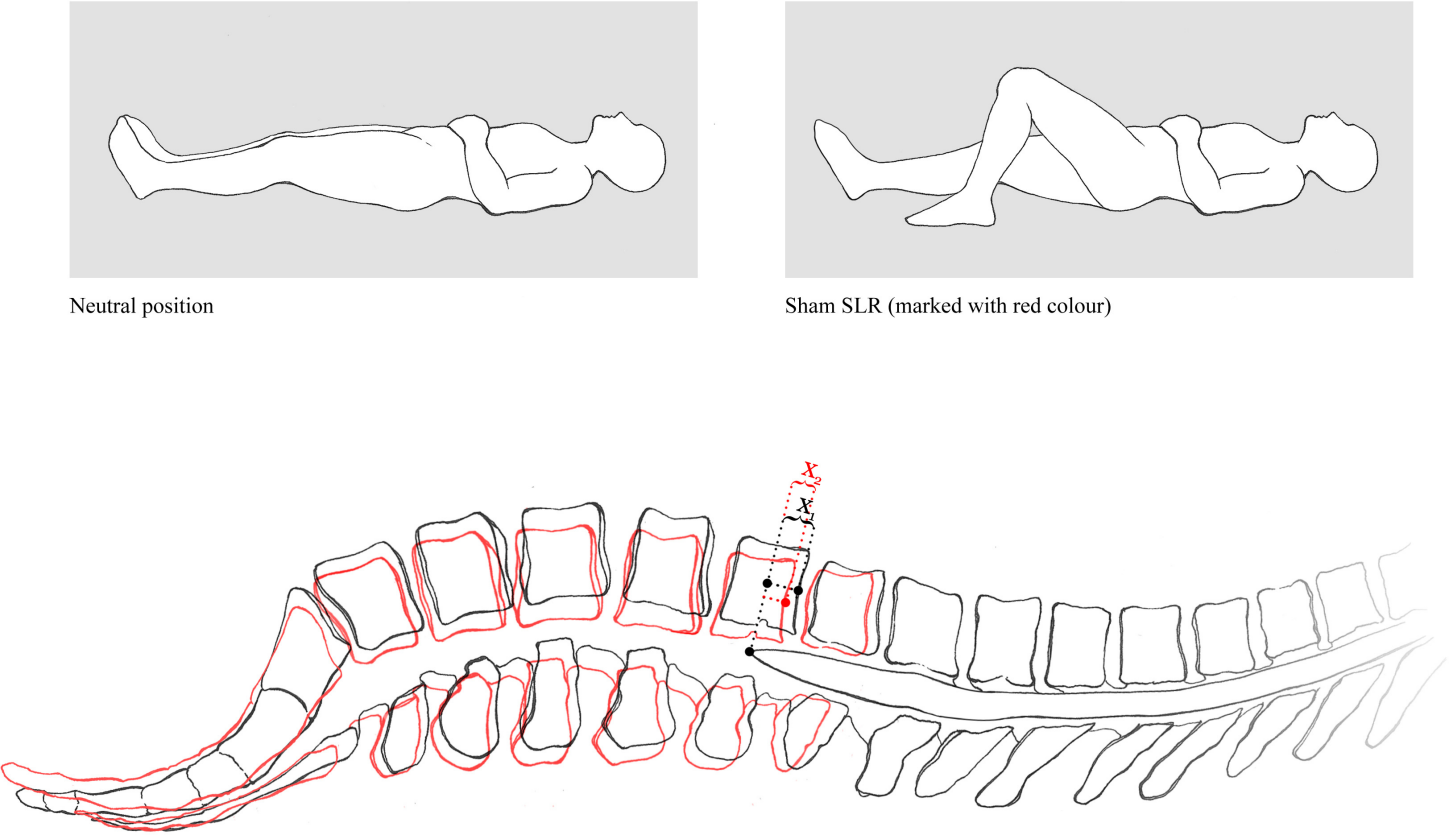
Lumbar lordosis flattens and the spinal canal adjusts around the static spinal cord following the pelvis movement in response to hip flexion. Note the motion of the vertebral bodies relative to the static conus medullaris during Sham SLR maneuver (red color), indicating free reciprocal motion between these two structures (X_1_>X_2_). It might be that the measured conus displacement during Sham SLR (0.32 mm) does actually indicate the magnitude of adaptation of the vertebral canal around the static conus. Image copyright: Ivan Barun, MD.

On a mechanisms level, this may be explained by the spinal cord remaining in its original position in the absence of tensile forces transmitted through the lumbosacral nerve roots and dura, while the spinal canal is adjusting around the spinal cord, as explained in Fig 6. It might be hypothesized that the conus displacement during Sham SLR actually indicates the magnitude of adaptation of the vertebral canal around the static conus, quantifiable in 0.32±1.6 mm. Following this, the conus displacement in response to unilateral SLR may be due both to i) transmission of tensile forces throughout the nerves and dura inducing free neural sliding and due to ii) reciprocal movements between the spinal cord and the surrounding vertebrae. These two components can be easily discerned by mathematical subtraction so that:

Mean unilateral SLR displacement–Mean sham SLR displacement=Mean conus displacement due to neural sliding

where values are expressed as absolute values.

So out of 3.54 mm of conus displacement (absolute values) recorded during standard unilateral SLR, 3.22 mm may be attributable to pure neural sliding relative to the adjacent vertebral body and 0.32 mm to adaptation of the vertebral canal around the conus in response to pelvis movement during a SLR (3.54mm– 0.32mm = 3.22mm).

These values would be summing up (3.54mm + 0.32mm = 3.86mm) if the reference point was located outside the subject’s body, as in that case both conus and vertebrae would move in the same direction relative to and external reference point. As it is possible that pain is provoked when there is a restricted range of motion of neural structures relative to their interface mostly due to tensile stress acting upon those neural structures, in our opinion it is more relevant and clinically meaningful to quantify the sliding of neural structures relative to their adjacent interfacing musculoskeletal structures. With these results, the authors expect that the sliding of neural structures in the vertebral canal may indeed represent a protective mechanism which preserves the spinal cord and neural roots from excessive strain. If this were true, the preservation of free sliding of the neural structures in the vertebral canal, along with the associated meninges, might indeed be an essential condition for maintaining an asymptomatic spine.

Moreover, on statistical power calculation emerged that 2621 subjects would be needed to be scanned to achieve statistical significance for conus medullaris displacement with a right Sham SLR, and 78 with a left one. This suggests that the observed power of the measured phenomenon is extremely low. From these results, it may be said that the magnitude of conus medullaris displacement with a Sham SLR is possibly irrelevant (<0.5mm).

The high correlation values presented in this study show that these movements in response to both Standard and Sham SLRs were consistent and reproducible but, as the magnitude and direction of the movements during the Sham SLR seem to be consistent within subjects, but not between subjects, it seems that they cannot be predicted generally.

Three relevant points emerge from the presented data: i) reciprocal movements between the spinal cord and the surrounding vertebrae are likely to occur during SLR, ii) conus medullaris displacement in the vertebral canal with SLR is primarily due to transmission of tensile forces through the neural tissues, iii) when tensile forces are transmitted through the neural system as in the clinical SLR, the magnitude of conus medullaris displacement prevails over the amount of bone adjustment.

The notion that during the clinical SLR the magnitude of conus medullaris displacement prevails over the amount of bone adjustment due to transmission of tensile forces throughout the neural system indicates that SLR is indeed prevalently a neural test.

Moreover, it might be that unrestricted reciprocal motion between neural and interfacing musculoskeletal structures inside the spinal canal and possibly foramina may be the conditio sine qua non for maintaining an asymptomatic spine [1,18,19]. Therefore possibilities relating to measurement of such displacement may have future diagnostic value. Efforts are currently taken by the authors of this study to investigate such responses in patients with radiologically proven intervertebral disc herniation causing sciatica.

Transmission of tensile forces along the sciatic nerve and L5 and S1 nerve roots with SLR has been shown to occur by Gilbert and colleagues [16]. In their experiment, SLR with ankle dorsiflexion was shown to increase tension in the L5 and S1 nerve roots compared to the genuine SLR.

They also quantified neural excursion during the unilateral SLR to be 0.48±0.55mm for L5 and 0.51±0.73mm for S1, which is much less that presented in our actual paper and in the existing literature. The authors’ explanation was that the lesser amount of neural root displacement was primarily attributed to the preservation of intact foraminal ligaments in the investigated cadavers, however it seems that further reasons for such differences may lie in i) the old age of the cadavers donors (average 78.2 years) which can warrant for age-related degenerative changes that may have altered the normal biomechanics of the investigated region, ii) the lack of full knee extension in two of the five explored cadavers and iii) the fact that pelvic and spinal motion were limited with lag bolts screwed into the Ilium bilaterally, which did not allow to replicate completely the clinical SLR [18]. The points i) and iii) were discussed also by Gilbert and colleagues in their paper.

Also Smith and colleagues [4] prevented pelvic motion while measuring lumbar nerve roots displacement with SLR in cadavers by fixing the pelvis with a metal spike driven through the anterior iliac wings and into the underlying wooden board. However, they presented results showing caudal sliding of 1.4 mm for L4, 2.1 mm for L5 and 2.5 mm for S1 nerve roots which is in agreement with previous works [6,7,9]. In this case, neural sliding may have been aided by their posterior unilateral laminectomies and facetectomies which may have impaired the function of foraminal ligaments.

A relevant notion that emerges from these two studies is that both foraminal ligaments integrity and natural pelvic motion should be preserved when attempting to replicate the clinical SLR performance in cadaver investigations. Performance of those investigations in in-vivo and structurally intact human subjects helps fulfill this requirement.

Intact foraminal ligaments and natural pelvic motion were maintained in our study. However, it is possible that hamstrings muscles might have had a different impact on pelvis movement between genuine SLR and Sham SLR. Unfortunately we were not able to quantify this effect as the MRI field of view was limited to the thoracolumbar spine. This point represents a possible limitation to our study and suggestion for future improvement.

## Conclusions

From the data presented in this investigation it emerges that i) reciprocal movements between the spinal cord and the surrounding vertebrae are likely to occur during SLR, ii) the conus medullaris displacement recorded with SLR is primarily due to transmission of tensile forces through the neural tissues, iii) when tensile forces are transmitted through the neural system as in the clinical SLR, the magnitude of conus medullaris displacement prevails over the amount of bone adjustment.

It appears that, even though both spinal canal adjustments and neural tension contribute, the neural tension mechanism dominates the process.

These results are similar to those presented by Breig in his cadaver investigations [20,21]. The correlation of our results with those in cadaver investigations supports the accuracy of the MRI methods employed in our earlier published studies [19] as well as this study.
